# Increasing deep-water overflow from the Pacific into the South China Sea revealed by mooring observations

**DOI:** 10.1038/s41467-023-37767-4

**Published:** 2023-04-10

**Authors:** Chun Zhou, Xin Xiao, Wei Zhao, Jiayan Yang, Xiaodong Huang, Shoude Guan, Zhiwei Zhang, Jiwei Tian

**Affiliations:** 1 Frontier Science Center for Deep Ocean Multispheres and Earth System (FDOMES) and Physical Oceanography Laboratory, Key Laboratory of Ocean Observation and Information of Hainan Province/Sanya Oceanographic Institution, Ocean University of China, Qingdao/Sanya, China; 2 Laoshan Laboratory, Qingdao, China; 3 Woods Hole Oceanographic Institution, Woods Hole, 02543 MA USA

**Keywords:** Physical oceanography, Physical oceanography

## Abstract

Cold and dense water from the North Pacific Ocean that spills through the Luzon Strait, the only deep conduit between the South China Sea (SCS) and the Pacific Ocean, renews deep-water mass, modulates hydrographic and biogeochemical cycles, and drives abyssal and overturning circulations in the SCS. The variability of this key oceanic process, however, has been poorly studied, mainly due to a lack of sustained observations. A comprehensive observational program that started in 2009 has provided 12 years of continuous time series of velocity and volume transport within the Luzon Strait. Here we show the observation-based assessment of decadal trends of deep-water transport through this vital passage. With the estimated 12-year mean volume transport of the deep-water overflow into the SCS of 0.84 ± 0.39 Sv (1 Sv = 10^6^ m^3^ s^−1^), a significant linear upward trend of 9% is revealed during this period. This is consistent with long-term changes in satellite-observed ocean bottom pressure. The results of this study may have broad implications for the overturning circulations and biogeochemical processes, including carbon cycles in this region.

## Abstract (web summary)

The observed deep-water overflow transport from the Pacific into the South China Sea has increased by 9% since 2009. This finding may have broad implications for the overturning circulations and biogeochemical processes in this region.

## Introduction

As the natural gateways for abyssal oceanic flows, deep passages, such as the Vema Channel, Romanche Fracture Zone, Denmark Strait, Samoan Passage, Yap-Mariana Junction, Atlantis II Fracture Zone, etc., are considered vital choke points where deep circulations around the globe can be effectively monitored. Exchange flows through those passages play a fundamental role in transporting not only heat and salt but also carbon and nutrients between deep basins and thus represent an important component in the global ocean circulation and biogeochemical cycle^1–9^. Observations at such choke points are often used to feel the pulse of abyssal flows and the lower limbs of meridional overturning circulations. For instance, Hansen et al.^9^ identified a warming and weakening trend of the Faroe Bank Channel overflow since 1950 and suggested a weakening Atlantic meridional overturning circulation (AMOC). In the South Atlantic Ocean, a decadal warming trend of the Antarctic Bottom Water through the Vema Channel based on data collected by 19 cruises was used to infer variability of abyssal pathways for the global thermohaline circulation^10^. In the South Pacific Ocean, Voet et al.^11^ compared mooring observations during 2012–2014 and 1992–1994 at the Samoan Passage and suggested that the deep water flowing through this major gateway, as an important element of the Pacific meridional overturning circulation, has been warming and weakening. However, most of these observations are not long enough to reveal multidecadal changes, and temporal gaps in observational records may also affect assessments and interpretations of shorter-term changes. Elsewhere observations have been maintained at key deep passages like Denmark Strait, Drake Passage, etc. Such observations, however, have been scarce in the North Pacific Ocean, leaving a big gap in our understanding of abyssal circulation and its variability there. In this study, we present analyses of the 12-year in situ observations acquired between 2009 and 2021 to investigate a key component of abyssal circulations in the North Pacific Ocean—the Luzon Strait Deep Overflow (LSDO).

**Fig. 1:**
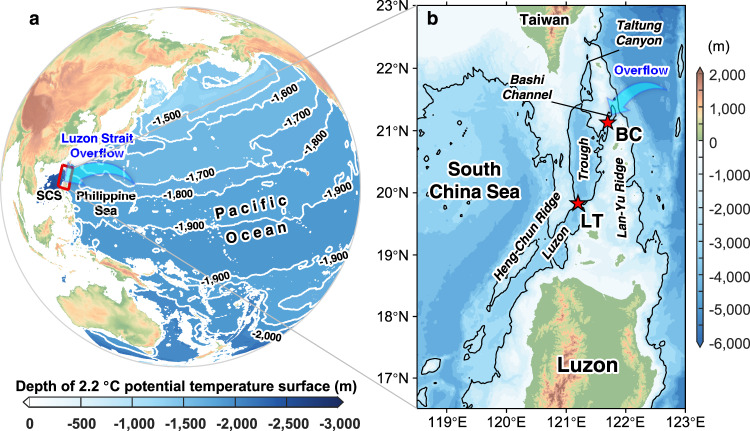
Location of the Luzon Strait. **a** Depth of 2.2 °C potential temperature surface in the Pacific, using updated data from the World Ocean Atlas 2013. **b** Enlarged map of the red box in (**a**) with topography from SRTM15+^69^ bathymetry. Red stars mark the mooring positions in this study. The black solid line represents the 2500-m isobaths. The bottom depth and mooring location are as follows. BC: 2720 m, 121.69 °E, 12.14 °N; LT: 3620 m, 121.20 °E, 19.83 °N. The abbreviation SCS in (**a**) represents the South China Sea. The abbreviations BC and LT in (**b**) represent BC mooring and LT mooring, respectively.

The South China Sea (SCS), a marginal sea with a deep basin over 1 × 10^6^ km^2^, is connected to the Pacific Ocean through Luzon Strait—a deep passage with a sill depth of ~2400 m. Due to the pressure difference between the deep Pacific Ocean and SCS^12^, the North Pacific Deep Water (NPDW) spills into the SCS, ventilates after going through vigorous mixing^13,14^, and is expected to exit the SCS in the upper layers (including the intermediate layer and the upper layer) through Luzon Strait and other shallow straits around the SCS^12,15,16^. The short residence time (30–70 years)^1,12,17^ of this cold and carbon-rich deep water suggests the SCS is a potential upwelling hotspot for the Pacific overturning circulation, making the deep-water overflow through the Luzon Strait, i.e., LSDO, an ideal indicator for the variability of the northwestern Pacific overturning circulation (Fig. 1a).

Since the late 20th century, the LSDO has been subject to increasingly more studies using direct observations, synoptic analysis, numerical simulations, etc. This has led to a better quantification of LSDO transport and the characteristics of its short-term variability. Based on an 80-day observation by a current-meter mooring, Liu and Liu^18^ presented the first direct evidence for the existence of LSDO. Subsequent analyses of longer time series of deep current observations^1,17,19,20^ provide a quantitative estimate of ~0.88 Sv for the mean volume transport of the LSDO. It has been postulated in previous studies^21^ that a considerable difference in deep-water mass density between the western Pacific Ocean and the SCS, which is likely the result of an enhanced mixing in the deep SCS^13^, drives the LSDO transport into the SCS. The seasonal changes of deep-water density contrast and, thus, the pressure difference between the Pacific and SCS also seem to explain the strengthening and weakening of LSDO transport in autumn–winter and spring–summer seasons, respectively^1,20^. In addition, intraseasonal variations with periods of ~30 days have also been observed and discussed^1,17,19,20^. Longer-term changes in LSDO transport, however, remain virtually unknown due to a lack of sustained observations. This study presents the first analyses of decade-long changes in LSDO based on direct observations that have been made right at Luzon Strait, filling a large gap in our understanding of a key ocean circulation component in the western Pacific Ocean that is known to play an important role in many aspects of climate and marine environments.

## Results

The deep Luzon Strait between Taiwan Island and Luzon Island is cut across by two generally north-south-oriented ridges, i.e., the Heng-Chun Ridge and Lan-Yu Ridge, respectively, with one deep trench named the Luzon Trough in the middle (Fig. 1b). Two gaps lying at the northern part of the Lan-Yu Ridge, the Taltung Canyon with a sill depth of ~2000 m and the Bashi Channel with a sill depth of ~2400 m, connect the Luzon Trough with the deep Western Pacific Ocean. The North Pacific Deep Water (NPDW) intrudes into the Luzon Trough mainly through the Bashi Channel and sequentially spills into the deep SCS through gaps across the Heng-Chun Ridge^17,20,21^.

In October 2009, two current-meter moorings, named BC and LT, were deployed at the saddle points of the Bashi Channel and Luzon Trough to continuously monitor the LSDO (Fig. 1b). Results from the first 3.5-year time series of the LSDO have been discussed by Zhou et al.^1^ (hereinafter, Z14) in details focused on the mean characteristics, intraseasonal and seasonal variabilities. The moorings were recovered and redeployed yearly to refurbish and maintain the instruments, as well as adjust the configurations of the moorings to better resolve the LSDO (Supplementary Fig. S1). As of the latest recovery in May 2021, almost 12 years of continuous record of the LSDO has been acquired.

### Mean-state and short-term variability of the LSDO

**Fig. 2:**
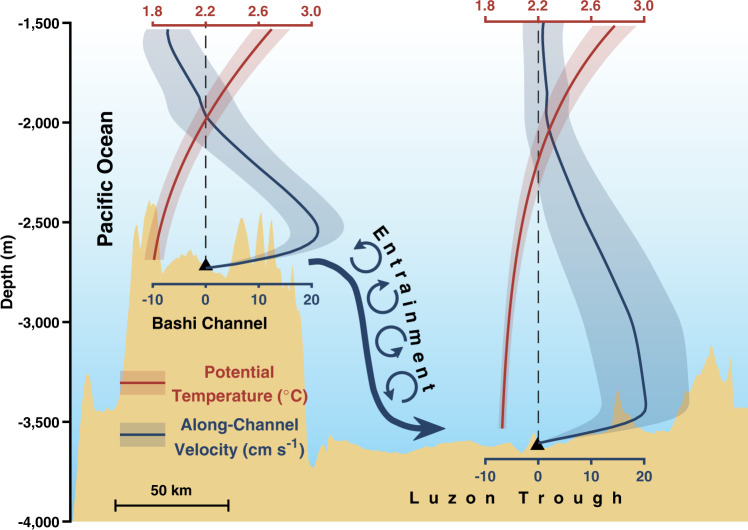
Mean state of the Luzon Strait overflow. The vertical profile below 1500 m of the whole-period-mean potential temperature (red) and along-channel velocity (blue) at BC and LT, respectively, with shading showing tide-excluded standard deviation. Positive (negative) velocity indicates that waters flow into (out of) the South China Sea. The black triangles indicate the mooring position. The black dashed lines denote zero velocity and a 2.2 °C profile. The abbreviations BC and LT represent BC mooring and LT mooring, respectively.

Previous hydrographic observations indicate that the deep water from the North Pacific with a potential density (*σ*_*2*_) up to 36.93 kg m^−3^ could spill into the Bashi Channel, accompanied by elevation of upstream isopycnal surfaces tilting toward the sill^22^. The spilled water forms a bottom-intensified deep-water flow along the Bashi Channel and Luzon Trough, about 150 km downstream, as depicted in Fig. 2 based on the mean state of the 12-year continuous observations below the 1500 m depth. The core mean of the along-channel velocity (*V*_*a*_) of 21.2 ± 5.0 cm s^−1^ and 20.3 ± 8.3 cm s^−1^ (mean ± s.d.) are recorded at depths of 2540 m at BC and 3420 m at LT, respectively. Small differences exist when comparing these results with those of Z14, presumably due to the impact of interannual variability on the mean velocity in two different analysis periods. By using appropriate interpolation methods, 0.69 ± 0.20 Sv and 0.84 ± 0.39 Sv are the reasonable mean state of the Luzon Strait overflow based on 12-year-long time series (See “Methods”).

**Fig. 3:**
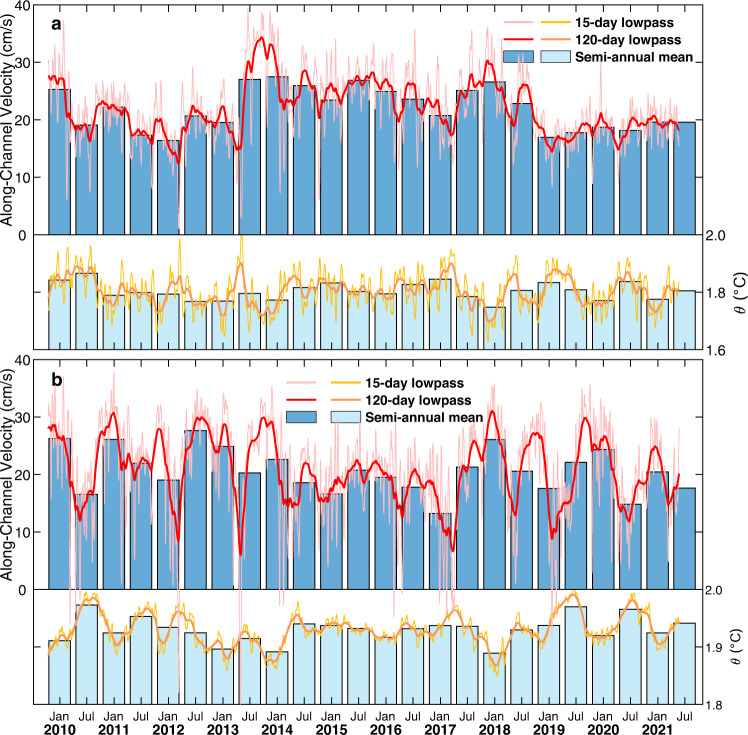
Variability of the Luzon Strait overflow. Fifteen-day lowpass (thin lines in pink and yellow), 120-day lowpass (thick lines in red and orange), and semi-annual mean (blue and cyan bars) series of along-channel velocity and potential temperature (*θ*) of the deepest instruments at **a** BC and **b** LT are shown, respectively. Positive (negative) velocity indicates that waters flow into (out of) the South China Sea. Semi-annual denotes winter half year (October–March in the next year) and summer half year (April–September). Noting that the last bar is April–June due to the length of observations. The abbreviations BC and LT represent BC mooring and LT mooring, respectively.

There is a noticeable difference in overflow variability between the two mooring sites at BC and LT (Fig. 3), possibly resulting from bathymetric effects on along-channel flows. Due to the rapid descending of bathymetry along the pathway of the overflow, the core of overflow deepens by ~880 m from BC to LT. Meanwhile, the water mass in the core of overflow experiences considerable warming from 1.85 ± 0.07 °C at BC to 1.93 ± 0.03 °C at LT and a decrease in density from 36.90 ± 0.01 kg m^−3^ at BC to 36.80 ± 0.01 kg m^−3^ at LT. The transformation of the overflow water mass between these two sections indicates a considerable mixing with overlying warmer and lighter water masses. The turbulent mass flux due to entrainment within a deep strait can be approximated by the average volume flow rate *Q* times the average decrease in density $\delta \rho$ observed between upstream and downstream^23^, which yields 1.8 × 10^4^ kg s^−1^ for the Luzon Strait. Previous studies have indicated that the enhanced mixing in the deep Luzon Strait^13,14^ may have been attributable to the energetic internal tides and their resonance between the two ridges^24^. It is estimated that the equivalent mixing rate over the deep SCS is (0.9–5.4) × 10^4^ kg s^−1^, which is comparable with the entrainment rate of the overflow (see “Methods”). Similarly strong entrainment of overflow has also been reported at the Samoan Passage^25^, Vemal Channel, Romache Fracture Zone^23^, and the Demark Strait^26,27^.

Observations made by the deepest moored instruments at BC and LT reveal distinct seasonal cycles of water temperature and velocity that are consequential to the seasonality of the mixing in the deep SCS^1^ (Supplementary Fig. S2). The variation range of tide-excluded (15-day lowpassed) velocity from a minimum of 1.1 cm s^−1^ to a maximum of 39.3 cm s^−1^ at BC and −20.3 cm s^−1^ to 37.8 cm s^−1^ at LT was surprisingly large (Fig. 3). Meanwhile, occasional reversals of the along-channel velocity were detected in boreal spring in both BC and LT, and the reversal flows were accompanied with positive temperature anomalies. This interesting phenomenon has also been reported in the Yap-Mariana Junction, Vema and Hunter Channels^28–30^. Mechanisms that are responsible for these overflow reversals are beyond the scope of this work.

### Long-term variability of the overflow

**Fig. 4:**
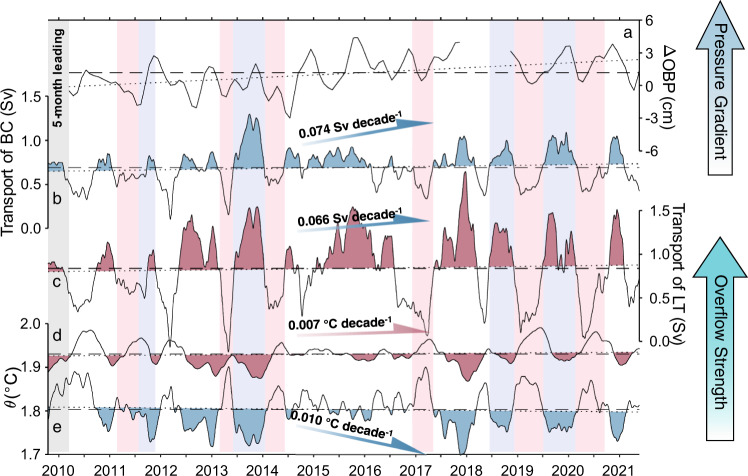
Trends of the Luzon Strait overflow. **a** Ocean Bottom Pressure (OBP) change from the Gravity Recovery and Climate Experiment (GRACE) between the South China Sea (110–120 °E, 10–20 °N) and the Pacific Ocean (122–130 °E, 16–24 °N) and observed transport and potential temperature (*θ*) time series of BC (in blue (**b**, **e**)) and LT (in red (**c**, **d**)), with shading showing overflow-enhanced periods, all series are 120-day lowpassed. Background color in light blue (red) indicates a strong (weak) pressure gradient with strong (weak) overflow. The dotted and dashed lines denote the linear trends and mean values of BC and LT, respectively. Positive (negative) transports indicate that waters flow into (out of) the South China Sea. Noting that ΔOBP series is moved 5 months forward for clear comparison. The abbreviations BC and LT represent BC mooring and LT mooring, respectively.

The 12-year dataset of continuous observations allows us to investigate interannual and decadal changes in the LSDO. Following the aforementioned interpolation method, the volume transport and potential temperature of the LSDO water mass at BC and LT from 2009 to 2021 are shown in Fig. 4. In addition to the substantial seasonal variability that was previously discussed by Z14, our in situ observations reveal a significant variability of LSDO on interannual time scales at both mooring sites. For 2009–2011 and 2016–2017, the averaged transport of LSDO was 0.61 ± 0.13 Sv (0.71 ± 0.20 Sv) and 0.61 ± 0.12 Sv (0.70 ± 0.36 Sv) at BC (LT), respectively, whereas, in 2012–2015 and 2018–2021, they were 0.74 ± 0.22 Sv (0.92 ± 0.40 Sv) and 0.73 ± 0.20 Sv (0.90 ± 0.45 Sv) at BC (LT), respectively. Interestingly, the periods with a strengthened (weakened) LSDO coincided with larger (smaller) standard deviations, indicating a stronger LSDO was accompanied by greater variability and vice versa.

In addition to interannual variations, noticeable decadal trends can be identified in the volume transport and potential temperature time series. Using the linear regression method, it is estimated that the decadal trends for volume transport were 0.074 ± 0.045 Sv (95% confidence interval, CI hereinafter, *p* < 0.01) and 0.066 ± 0.059 Sv (95% CI, *p* < 0.05) per decade at BC and LT, respectively. The potential temperature (*θ*) from the bottom CTD at BC and LT also display linear trends of −0.010 ± 0.008 °C (95% CI, *p* < 0.02) and 0.007 ± 0.008 °C (95% CI, *p* < 0.1) per decade during this 12-year observation period. These trends can also be demonstrated by the time series of linear trend of transport and temperature (Supplementary Fig. S3), which manifests the increment of the transport at BC and LT and the cooling at BC.

The cooling of the LSDO water mass at BC was accompanied by an increasing transport, both of which indicate a strengthening intrusion of relatively cold NPDW. However, a weak warming was detected at LT in contrast to cooling at BC, which is likely due to mixing that occurs between two mooring sites. Before arriving at LT, NPDW experiences vigorous entrainment with warmer ambient waters in the intermediate and upper layers (Fig. 2). Mixing with warm ambient water in the intermediate layer results in an increase of the mixed water temperature in the LSDO at the LT site.

The negative baroclinic westward pressure gradient is formed due to the cross-strait density gradient between the deep Pacific Ocean and SCS. Previous studies have indicated that the LSDO is directly driven by the pressure gradient between the deep Pacific Ocean and SCS^12,21^, whose variability could therefore lead to changes in the LSDO transport, such as seasonal changes^1^. However, the relationship between the LSDO transport and the Pacific-SCS pressure gradient has not been rigorously tested against direct observations. The launch of the Gravity Recovery and Climate Experiment (GRACE) mission^31^ provides unprecedented long-term gravimetric observations of the ocean bottom pressure (OBP), which detects changes of ocean water mass above the seafloor and has been widely used for studies of long-term variability of deep ocean circulation^32–34^. Our decade-long observations, together with GRACE OBP data, provide a unique opportunity to test the often-cited relationship between LSDO transport and Pacific-SCS pressure difference. We calculate the OBP averaged in two domains, one to the east (122–130 °E, 16–24 °N) and another to the west (110–120 °E, 10–20 °N) of Luzon Strait. The difference shows a noticeable increasing trend during the period of our observations from 2009 to 2021 (Supplementary Fig. S4). This is consistent with our assessment that the LSDO transport of NPDW into the SCS has increased. Our analyses also indicate that variations in the Pacific-SCS OBP difference lead to changes in LSDO transport by 5 months. We postulate that this is the adjustment time scale that the LSDO responds to changes in the pressure difference. Similar relationships are reported at the Faroe Bank Channel; the variability of overflow in the Faroe Bank channel is well correlated with the variability of the pressure difference between both ends of the channel^35–37^. The increase in the LSDO transport may have started well before our mooring program that started in 2009. In fact, data from GRACE indicate that the OBP difference has indeed increased since 2002, when the GRACE mission started (Supplementary Fig. S4a).

This pressure difference (Δ*P*_Grace_), which could be inferred directly from GRACE data, between both sides of the LS includes a barotropic component (Δ*P*_trop_) related to the sea level and a baroclinic component (Δ*P*_clin_) associated with water density variations, i.e., Δ*P*_Grace_ = Δ*P*_trop_ + Δ*P*_clin_. By combining ‘geostrophic control’^38^ for the upper layer and the ‘hydraulic control’^39^ for the lower layer, Song^40^ proposed a two-layer model to estimate the deep volume transport through passages using OBP and sea level anomaly (SLA) data. We compute the difference of the averaged SLA between the two basins on the two sides of Luzon Strait using satellite altimetry since 2002 (Supplementary Fig. S4b). In contrast to the OBP, the SLA difference exhibits a weak decreasing trend in the past 20 years, which implies that the upward trend in the OBP must be due to an increase in water density difference. Repeated occupied CTD sections at 137 °E during 1990–2020 suggest a noticeable lightening of waters above 1000 m^41^ and warming of deep waters below 3000 m^42^, but the cooling of the NPDW spreading westward into the northern Philippine Basin^42^, which could contribute to the increase in density difference and therefore motivate the increasing LSDO. However, synchronous and continuous observations on two sides of the Luzon Strait are necessary to verify this conjecture and further clarify the dynamics.

## Discussion

The LSDO is the sole source that provides the carbon- and nutrient-rich deep-water mass into the SCS; therefore, it plays a pivotal role in the biogeochemical processes of the deep SCS^8,43–47^. This study presents analyses of a 12-year continuous observation of the LSDO transport—a measure of the NPDW intrusion into the SCS. The mean velocity at the core of LSDO is about 21.2 ± 5.0 cm s^−1^ and 20.3 ± 8.3 cm s^−1^ at BC and LT, respectively. The volume transport across these two sections is about 0.69 ± 0.20 Sv and 0.84 ± 0.39 Sv, respectively, which agree with previous observational studies^1,19,20^. The LSDO water mixes with ambient water when passing the Bashi Channel, which results in an increase in volume transport from BC to LT and a decrease in water mass density. It is estimated that the mixing has led to an entrainment of 1.8 × 10^4^ kg s^−1^, which is comparable with the equivalent mixing rate over the deep SCS.

Noticeable decadal trends are detected in the volume transport, about 0.074 ± 0.045 Sv and 0.066 ± 0.059 Sv per decade at BC and LT sites, respectively. The 9 ± 5% increase in transport of the cold NPDW was accompanied by a cooling of −0.010 ± 0.008 °C per decade at BC. These changes are non-ignorable in the deep ocean. Voet et al.^11^ find dense water through Samoan Passage decreased by 4% per decade with 0.010 °C per decade warming compared to measurements two decades earlier. Warming of dense water at a rate of 0.028 °C per decade was reported in the Vema Channel using repeat occupations of hydrographic sections^10^. The observed changes in LSDO transport are consistent with what is inferred from satellite measurements of the OBP from the GRACE mission in the last 20 years. Our analysis indicated that the strengthening of the LSDO transport had started no later than 2002 when the GRACE mission started. In addition to decadal trends, our in situ observations reveal the variability of LSDO on intraseasonal to interannual time scales at both mooring sites (Fig. 4), such as transport weakening in 2009–2011 and 2016–2017 and strengthening in 2012–2015 and 2018–2021. This interannual variability has not been reported in previous studies, and its origin is still unknown. While it is beyond the scope of this study to identify forcing mechanisms for such variations, our analyses would hopefully motivate further process and mechanism studies.

Our analyses of changes in LSDO have several implications for processes in both the western Pacific Ocean and SCS. First, the decadal trends in transport are clearly consistent with changes in the OBP but not with that in SLA (Supplementary Fig. S4b), suggesting that changes in water density may have been culpable for the strengthening of LSDO. Second, the deep SCS circulation forms largely from the planetary vorticity flux through Luzon Strait^48,49^. As suggested by the model simulations presented by Zhou et al.^50^ and Gan et al.^51^, the deep circulation in the SCS may have strengthened due to an increase in LSDO transport. Consequent changes of the stratification in the SCS are expected to affect internal waves^52^ and corresponding diapycnal mixing^53^. This is an interesting topic worth to be further investigated. Third, the energetic upwelling of *O* (10^−6^ m s^−1^) of bottom water in the SCS^1,12^ should be strengthened with an increment of (0.7 ± 0.4) × 10^−7^ m s^−^^1^ during 2009–2021. This suggests that the residence time of deep water below 2000 m, averaged to be ~50 years during 2009–2021, would decrease by the rate of 4 years per decade. Since the vertical motion dynamically links the circulation in upper and deep layers^54^, this would affect the overturning of the deep water in the SCS consequently, which has potential impacts on the biogeochemical processes in the SCS^8,45,55–57^, the thermohaline structure and variability of the Indonesian Throughflow and the meridional overturning circulation in the western Pacific^16,58–60^. However, it is noteworthy that there are vigorous multiscale processes in overflows elsewhere^61,62^, and tidally modulated mixing is reported in Luzon Trough^63^, and the observation is only available for its first decade. It remains uncertain how these processes impact changes in LSDO and whether the observed changes represent a long-term trend or multidecadal variations, e.g., Pacific Decadal Oscillation (PDO). These uncertainties demonstrate the importance of promoting and sustaining this monitoring program for the LSDO.

## Methods

### Data and processing

Temperature and salinity were measured by the SBE 37-SM conductivity–temperature–depth (CTD) recorders from Sea-Bird Electronics. The accuracies of the instruments are 0.002 °C for temperature, 0.003 mS cm^−^^1^ for conductivity, and 0.1% of the full-scale range for pressure (~7 m for CTDs in this study). Velocity was measured by Seaguard recording current meters (RCMs) from Aanderaa Instruments and Workhorse Long Ranger 75 kHz and 300 kHz acoustic Doppler current profilers (ADCPs) from Teledyne RD Instruments. The CTDs were sampled every 30 min, the RCMs every 60 min, and the ADCPs every 60 min. The accuracy of the velocity measurements is 0.15 cm s^−^^1^ for the Aanderaa RCMs and 0.1% *S* ± 5 mm s^−^^1^ for ADCPs (*S* stands for the water velocity relative to ADCP). Velocities exceeding five times standard deviations are precluded. Every CTD was calibrated or contrasted with the standard CTD before deployment. For CTDs with salinity drifts, the mean salinity of CTDs before and after their observational period is used. For instruments measuring only temperature (see Supplementary Fig. S1b before March 2010), salinities were estimated using mean values of corresponding months in the next year. Cubic spline interpolation is applied for filling gaps (generally a couple of hours) of all instruments due to deployment and recovery. Details on the mooring configurations are shown in Supplementary Fig. S1.

World Ocean Atlas 2013 (WOA13) objectively analyzed climatologies of temperature and salinity data^64,65^. These data were used to estimate the mean depths of isotherms and isopycnals in the Pacific Ocean and SCS (Fig. 1a). The WOA13 data used in this study is the average of six decades with 0.25° grids.

Ocean bottom pressure (OBP) anomalies are from the Gravity Recovery and Climate Experiment (GRACE), which was launched in 2002^31^ and ended in late 2017, and the follow-on mission was launched in 2018. Here we compare data products from all three GRACE processing centers, i.e., the Center for Space Research (CSR), the Helmholtz Centre Potsdam, German Research Center for Geosciences (GFZ), and the Jet Propulsion Laboratory (JPL), and differences among these products are small. For our analysis, we use monthly OBP anomalies derived from the new CSR-RL06v4M. Gaps in OBP anomaly time series are filled with cubic spline interpolation except from July 2017 to May 2018. The 1/4° gridded daily altimeter and sea level anomaly (SLA) from AVISO (Archiving, Validation, and Interpretation of Satellite Oceanographic) during the observation period were used to identify the sea surface height trend in the SCS and the Pacific Ocean.

### Calculation of volume transport

Analysis of a densely spaced mooring section deployed at the Bashi Channel suggested that one mooring in the middle of the channel, accompanied by a proper interpolation method, could arrive at a fairly accurate estimate of volume transport of the deep-water overflow due to the narrow topography constraint^19^. Transports of LSDO were calculated by interpolating the mooring currents perpendicular to the direction of the along-channel velocity (*V*_*a*_, which is 230° and 201° clockwise from north at BC and LT, respectively) onto a grid with a horizontal resolution of about 0.1 km and a vertical resolution of 20 m for every time step (1-day interval). On the basis of our mean velocity profiles from the moored ADCPs, we find velocities to decrease toward the seafloor and the upper layers. We choose the level of no motion based on the mean profile of along-channel velocity in Fig. 2 at BC. The velocity at 1920 m of LT is small (~1.4 cm/s) compared with the core velocity (~20 cm/s), and the velocity does not change considerably if we choose the upper boundary between 1900 and 2000 m. Assuming that the along-channel velocities are zero at two boundaries of the strait and using bathymetry in the inset of Supplementary Fig. S1, we interpolate the velocity in cubic spline horizontally and in linear vertically, by using different interpolation methods giving uncertainties of 0.19 Sv and 0.20 Sv at BC and LT, respectively. For lack of instruments at the beginning of the observation, we linearly extrapolated the velocity to the level of no motion of BC at 1920 m, which brings 0.11 Sv and 0.08 Sv uncertainties at BC and LT, respectively, compared with the mean velocity profile using more instruments after 2012. The volume transport is calculated from one or two current meters. Noting that the mooring located at LT from July 2015 to July 2017 is not exactly at the deepest site of the channel, which affects 0.017 Sv of the mean transport at LT. The uncertainty in the current measurement (0.15 cm s^−^^1^) results in errors of 0.009 Sv and 0.015 Sv at BC and LT, respectively. All these uncertainties are within one tide-excluded standard deviation, which is 0.20 Sv and 0.39 Sv at BC and LT. As a result, 0.69 ± 0.20 Sv and 0.84 ± 0.39 Sv (mean ± s.d.) are considered to be the reasonable mean state of the Luzon Strait overflow based on 12-year-long time series.

### Linear regression

The linear trend was estimated by applying a one-dimensional linear regression model in the least-squares sense to explore the long-term variability of the LSDO. The *F*-statistic is used to test the significance of the regression model. Monte Carlo simulation with block bootstrapping^66,67^ is used to quantify the significance of the trends. Specifically, for a certain observed time series, we first (1) calculate the block length according to the effective decorrelation time *τ*^67^, and then (2) randomly resample with replacement blocks of length τ from the observed time series and concatenate them to create a time series with the same length of the observed one. Next (3), we conduct the regression of the series. After 1000 random simulations (*B* = 1000), sorting the fitted parameters (slope), the 95% (90%) confidence interval follows from the 25 and 975 (50 and 950) fitted values. This process is repeated 100 times (*I* = 100) to get average slopes. Meanwhile, effective degrees of freedom (DOF) could be verified using *N*_eff_ = *N*/*τ*. By comparing the slope of each regression between the real (*β*) and randomized (*β*_b,i_, b = 1, 2,…, *B*; i = 1, 2, …, *I*) dependent variable, we obtain a frequency (i.e., the number of simulations have absolute values |*β*_b,i_ | larger than |*β* | ) from each run, and then divide the count by 1000 to get the *p*-value (*p*_i_, i = 1, 2, …, *I*). After 100 iterations, we could obtain the averaged *p*-value. Most trends are statistically different from zero (*p* < 0.05), while the potential temperature trend at LT is not (*p* < 0.1). For time series (OBP and SLA) with low auto-correlation (smaller *τ*), we still use *F*-test statistics to obtain the *p*-value.

### The turbulent mass flux due to entrainment

The turbulent mass flux due to entrainment within a deep strait can be approximated by the average volume flow rate *Q* times the average decrease in density $\delta \rho$ observed between the upstream and downstream^68^. For the Luzon Strait, *Q* = 0.69 Sv, which is the transport at BC, and $\delta \rho \cong 0.026kg\,{m}^{3}$ based on the potential density of the core of overflow at BC and LT, which yields $Q\delta \rho \cong \,1.8\times {10}^{4}\,{{{{{{\rm{kgs}}}}}}}^{-1}$. Considering the reported strong diapycnal mixing in the deep SCS, where *κ* ~ 10^−^^3^ m^2^ s^−1^ ^14,21^, the average potential density gradient below 2000 m, $d\rho /dz=(0.9-5.4)\times {10}^{-5}\,{{{{{{\rm{kgm}}}}}}}^{-4}$, from WOA dataset and the area of the deep SCS basin ${A}_{{{{{{\rm{SCS}}}}}}}=1.0\times {10}^{6}\,{{{{{{\rm{km}}}}}}}^{2}$ at 2000 m, we have${A}_{{{{{{\rm{SCS}}}}}}}\kappa (d\rho /dz)=(0.9-5.4)\times {10}^{4}\,{{{{{{\rm{kgs}}}}}}}^{-1}$.

### Residence time and mean upwelling rate

Given the fact that the Luzon Strait is the only deep passage below 2000 m, by assuming a closed mass budget, the average residence time of waters in the SCS can be approximated by dividing the SCS volume by the estimated time-mean transport. Similarly, the average upwelling rate of waters in the SCS can be approximated by dividing the SCS area by the estimated time-mean transport. The long-term variability of residence time and upwelling rate are calculated in the same way by the estimated trend of LSDO transport. The volume and area of the SCS have been computed on the basis of SRTM15+ topography^69^, which are 1.3 × 10^6^ km^3^ below 2000 m and 1.0 × 10^6^ km^2^ at 2000 m, respectively.

## Supplementary information

Supplementary Information

## Data Availability

The global bathymetry and topography at 15 arcseconds (SRTM15+) data are downloaded from https://topex.ucsd.edu/WWW_html/srtm15_plus.html. Sea level anomalies are downloaded from CMEMS (http://marine.copernicus.eu/). The WOA hydraulic data are from https://www.nodc.noaa.gov/OC5/woa13/. The GRACE OBP data can be accessed at https://podaac.jpl.nasa.gov/. The data used for plotting the figures in the paper are available from 10.5281/zenodo.7748802.

## Code availability

The code used for plotting the figures in the paper can be accessed at 10.5281/zenodo.7748802.
