# Supplementary material for: Increasing deep-water overflow from the Pacific into the South China Sea revealed by mooring observations
Source: Nat Commun. 2023 Apr 10;14:2013. doi: 10.1038/s41467-023-37767-4 (PMC10085979; doi:10.1038/s41467-023-37767-4)
Supplement: Supplementary file 1 — Supplementary Information [file 41467_2023_37767_MOESM1_ESM.pdf]

Supplementary Information for

Increasing deep-water overflow from the Pacific into the South China Sea revealed by mooring observations

Chun Zhou, Xin Xiao, Wei Zhao*, Jiayan Yang, Xiaodong Huang, Shoude Guan, Zhiwei Zhang, Jiwei Tian*

*Corresponding author. weizhao@ouc.edu.cn (W.Z.); tianjw@ouc.edu.cn (J.T.)

The supplementary information includes:

Figs. S1 to S4

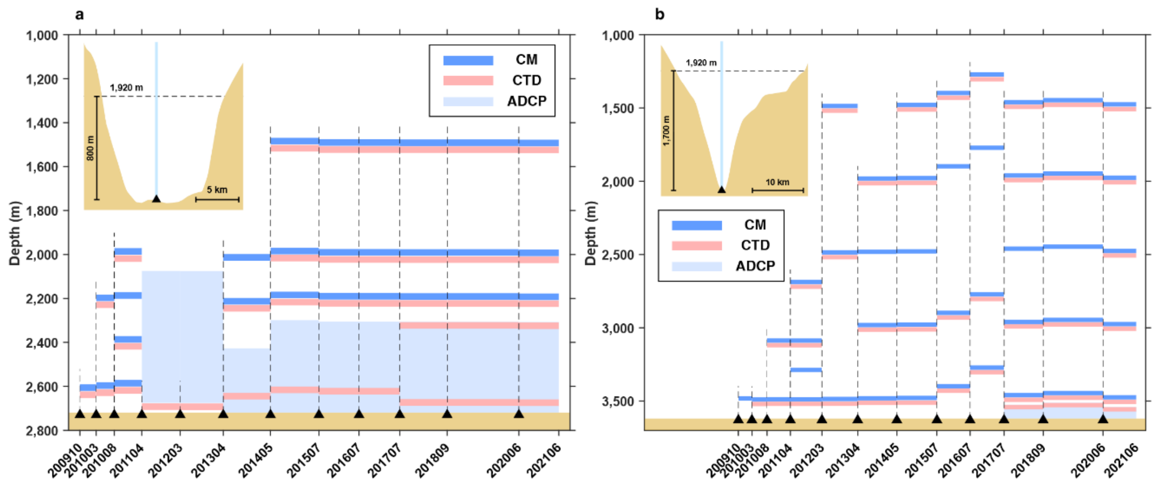

Fig. S1. Overview of the configuration of moorings. Instruments of (**a**) BC and (**b**) LT are shown respectively during 11.7-year observations. Duration of Acoustic Doppler Current Profiler (ADCP) and Current Meter (CM) are shaded in light blue and blue, respectively. Duration of Conductivity–Temperature–Depth (CTD) is shaded in light pink. Dotted lines denote time when the moorings were refurbishments. The section perpendicular to the mean overflow core, which is used to calculate volume transport is shown in the inset. Cyan bold line and the triangle in black indicate the mooring position cross the channel. The height above bottom of 1,920m-interface is shown in the inset. Bottom topography shaded in brown. The abbreviations BC and LT represent BC mooring and LT mooring, respectively.

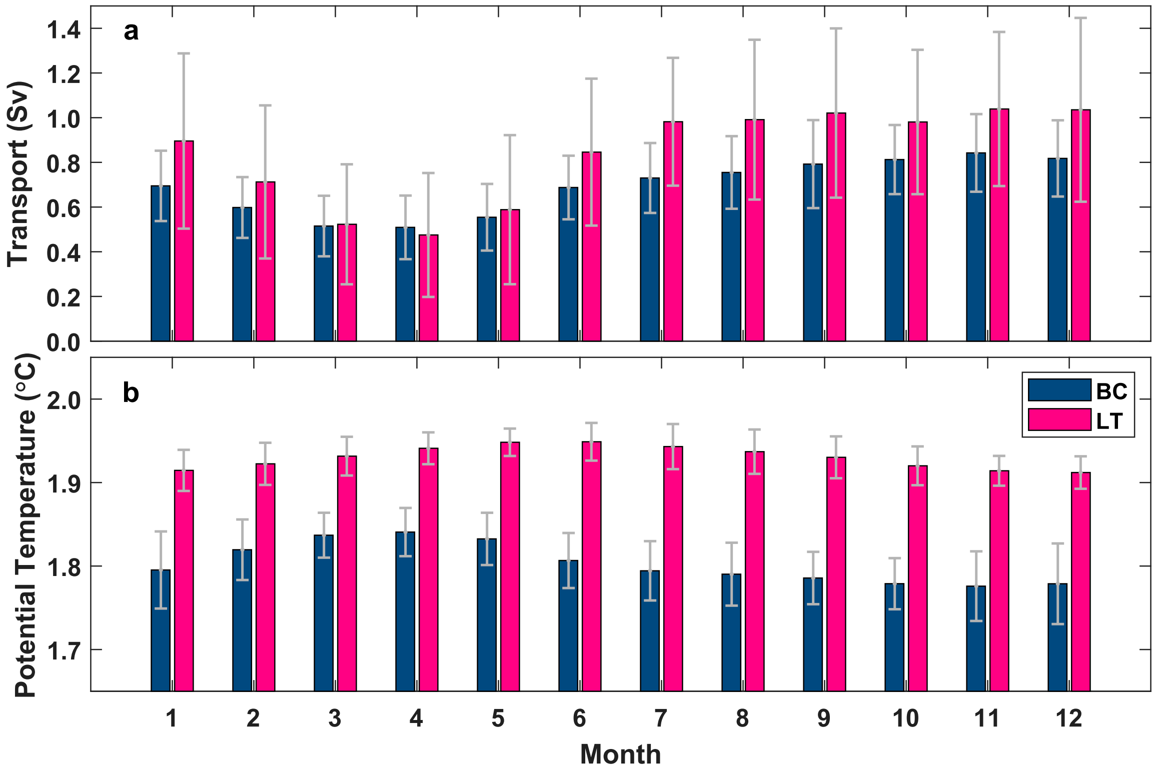

Fig. S2. Seasonal variability of the Luzon Strait overflow. Mean seasonal cycle of (**a**) transport and (**b**) potential temperature at the BC and LT based on monthly averaged time series. Tide-excluded standard deviation deviations are indicated by gray bars. The abbreviations BC and LT represent BC mooring and LT mooring, respectively.

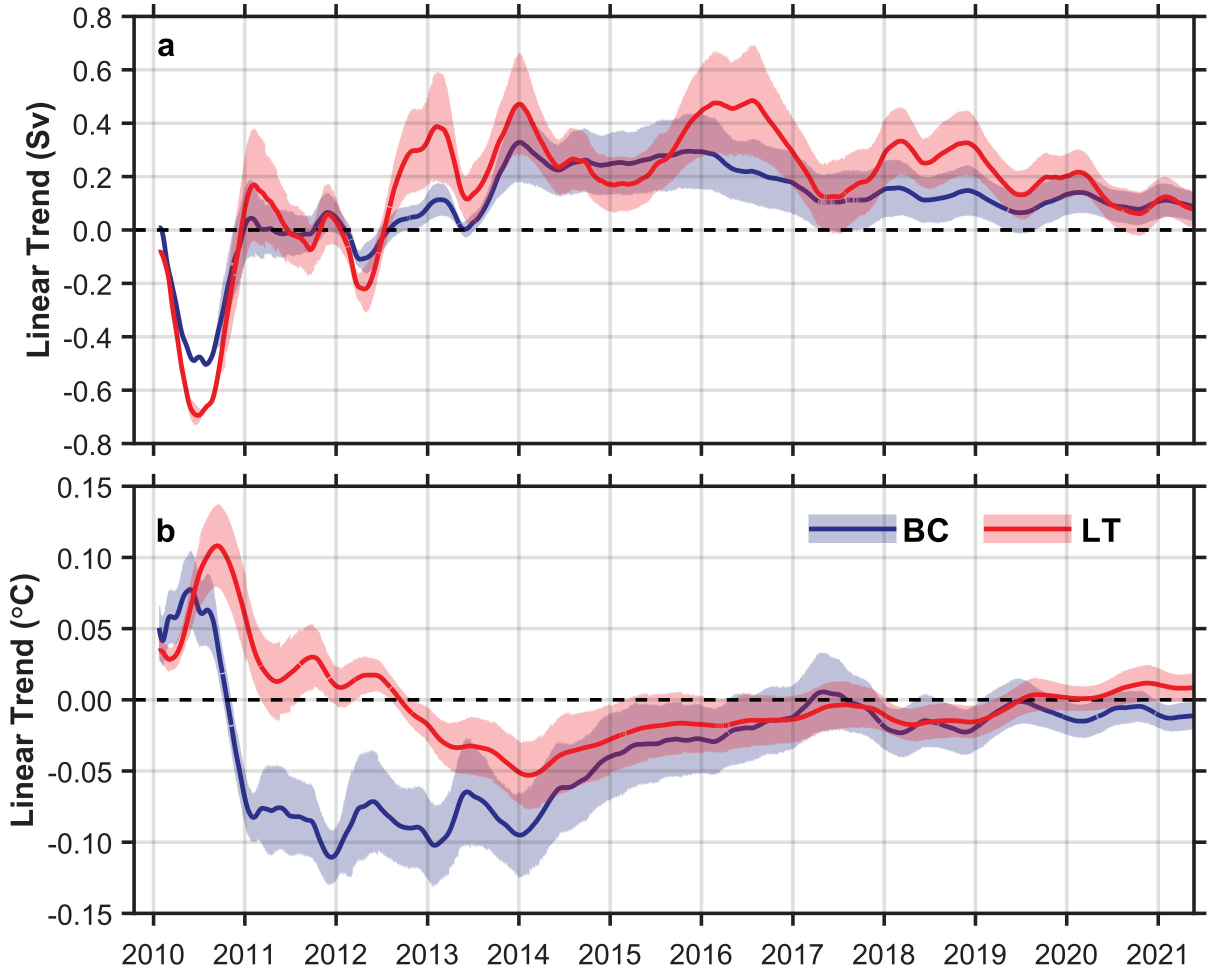

Fig. S3. Time series of linear trend of the Luzon Strait overflow. Trends of observed 120-day lowpass (**a**) transport and (**b**) potential temperature at the BC and LT since moorings deployed, with shading showing 95% confidence intervals using block Monte Carlo methods. Note that only results after Jan 2010 presented, to avoid uncertainties due to short length of time series. The abbreviations BC and LT represent BC mooring and LT mooring, respectively.

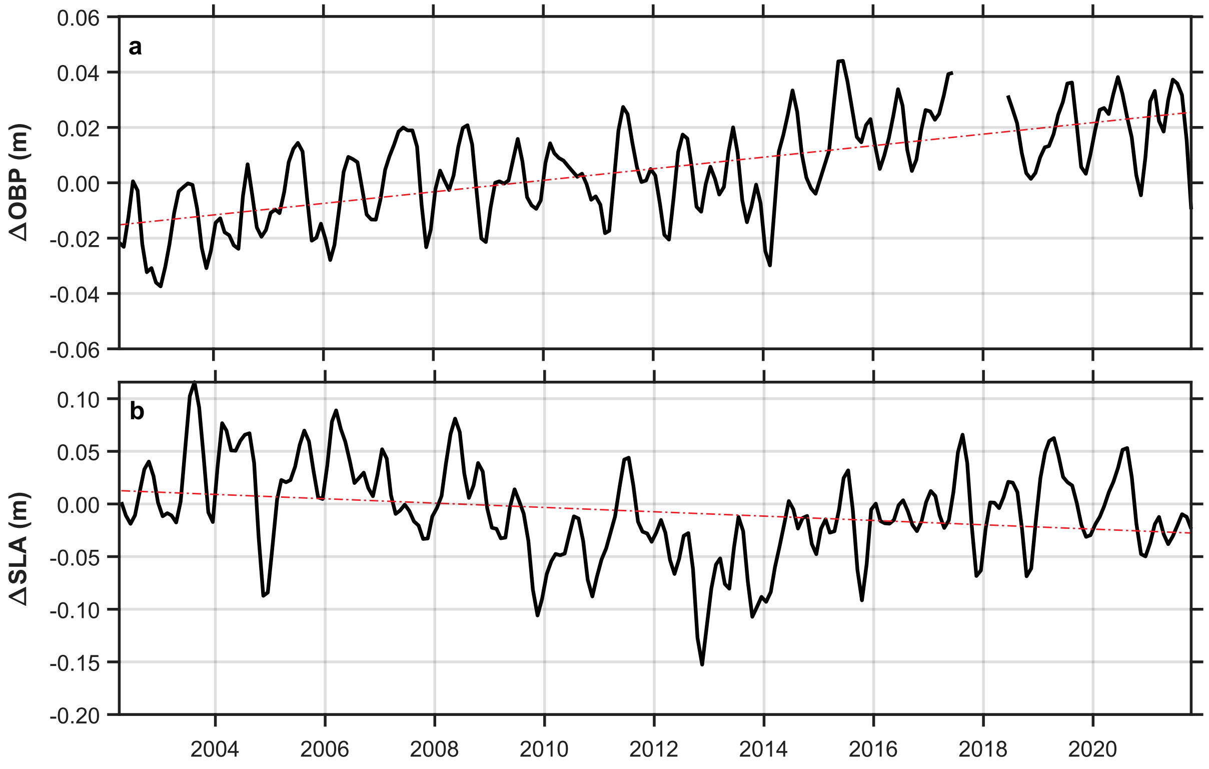

Fig. S4. Changes of Ocean Bottom Pressure (OBP) and Sea Level Anomaly (SLA). Time series (black solid line) and the trend (red dotted line) of (**a**) OBP (F=174, DOF=222, p<0.01) and (**b**) SLA (F=13, DOF=233, p<0.01) gradient between the South China Sea (110-120 °E, 10-20 °N) and the Pacific Ocean (122-130 °E, 16-24 °N).
